# Ultrastructure of the fertilized egg envelopes in *Ancistrus cirrhosus*, Loricariidae, Teleostei

**DOI:** 10.1186/s42649-020-00034-7

**Published:** 2020-06-17

**Authors:** Dong Heui Kim

**Affiliations:** Department of Environmental Medical Biology, Yonsei University Wonju College of Medicine, Wonju, 26426 South Korea

**Keywords:** *Ancistrus cirrhosus*, Egg envelope, Fertilized egg, Loricariidae, Ultrastructure

## Abstract

We examined the morphology of fertilized egg and ultrastructures of fertilized egg envelopes of *Ancistrus cirrhosus* belong to Loricariidae using light and electron microscopes. The fertilized eggs formed a mass on the spawning place and were yellowish, spherical, non-transparent, demersal, adhesive, and a narrow perivitelline space. But, the adhesiveness of fertilized eggs was disappeared after spawning excluding contact parts. The micropyle with funnel shape was surrounded by 15–19 furrow lines of egg envelope in a spoke-like pattern. The outer surface of egg envelope has smooth side and inner surface of egg envelope was rough with grooves. Also, the total thickness of the fertilized egg envelope was about 32.58 ± 0.85 μm (*n* = 20), and the fertilized egg envelope consisted of three layers, an outer adhesive electron-dense layer, a middle layer with low electron density and an inner electron-dense layer with grooves in counter structure from other most teleost. Collectively, these morphological characteristics and adhesive property of fertilized egg, and ultrastructures of micropyle, outer surface, and section of fertilized egg envelope are showed species specificity.

## Introduction

Jumbie teta (*Ancistrus cirrhosus* Valenciennes, 1836) is a teleost belong to Loricariidae, Siluriformes, and Actinopterygii. This species inhabits in Parana River basin of Argentina and Uruguay and algae-eater (Fishbase contributors 2020). A male guard eggs and larvae for up to 10 days after hatching in a cavity nest. It is known to female *Ancistrus* preferentially spawn with males guarding larvae, and the male’s snout tentacles stimulate this bias by mimicking the presence of larvae in an otherwise empty nest (Sabaj et al. 1999).

In teleost, the fertilized egg is surrounded by an acellular egg envelope, this envelope commonly is referred to as chorion, external membrane, capsule, outer envelope, egg shell, zona radiata, zona pellucida according to the researchers (Hisaoka and Battle 1958; Bell et al. 1969; Anderson 1974; Dumont and Brummet 1980; Grierson and Neville 1981; Schmehl and Graham 1987; Hamazaki et al. 1989). The egg envelope plays a role in diffusive exchanges of gases such as O_2_ and CO_2_, selective transport of necessary materials into the egg, protection of providing physical impact, chemicals and pathogens, fixation to a spawning ground in case of adhesive type, and inhibition of polyspermy through micropyle, sperm entry part (Grierson and Neville 1981; Laale 1980; Harvey et al. 1983; Cameron and Hunter 1984).

In teleost, the structure of fertilized egg and egg envelope has been known to be related with physical and chemical properties of water environment, and geographical distribution (Ivankov and Kurdyayeva 1973; Stehr and Hawkes 1979; Laale 1980). Also, the morphology of fertilized egg and ultrastructure of outer surface, micropyle, and section from fertilized egg envelope were showed species, genus or family specificities (Kim et al. 1998, 1999; Joo and Kim 2013; Kwon et al. 2015; Choi et al. 2019).

Some species belong to genus *Ancistrus* were studied on the cytogenetic diversity and the evolutionary dynamics of rDNA genes and telometic sequences (Favarato et al. 2016), spawning related with snout tentacles as a novel reproductive strategy (Sabaj et al. 1999) and chromosome polymorphism in *Ancistrus cuiabae* Knaack (Mariotto et al. 2009). But there is no study on the ultrastructure of fertilized egg envelope because it is hard to get fertilized eggs from this species in aquarium. So, we studied the morphology of fertilized egg, and the ultrastructures of micropyle, outer surface, inner surface and section of fertilized egg envelopes under the light and electron microscopes to find out species specificity in Jumbie teta, *Ancistrus cirrhosus* belong to Loricariidae, Siluriformes, and Actinopterygii with special spawning behavior.

## Materials and methods

### Animals

A pair of Jumbie teta, *Ancistrus cirrhosus* (total length: 10–12 cm) used in this study were purchased from SanHo Aquarium (Wonju, Korea). The tap water used for rearing was treated with carbon filter (Pre-carbon filter, filter114 Co. Ltd., Korea) to remove chlorine, and its temperature and pH were maintained at 26 ± 0.5 °C and 7.0 ± 0.5, respectively. Biological filtration was performed using a sponge filter (Tetra TwinBrillant Super Filter™, Tetra Co. Ltd., Germany), and scraps and excrement settled to the bottom of the water tank were eliminated by exchanging one-third of the water every day. The artificial light was illuminated for 9 h per day to simulate a daytime environment using an electronic timer, and raw green pumpkin and Brine Shrimp Plus Flakes™ (Ocean Nutrition, U.S.A.) were provided as food two times per day at 9 a.m. and 4 p.m.

### Collection of fertilized eggs

The water of breeding glass water tank (45X30X30 cm) was made by mixing rearing water and purified water by reverse osmosis, then was treated with peat moss for cultivation. It was adjusted to 25 ± 0.5 °C, 80 ppm, and pH 6.0 ± 0.5, respectively. The flower clay pot was used as a spawning ground. The fertilized egg mass was isolated using stainless chopsticks being careful not to break the fertilized eggs. Fertilized eggs which confirmed the formation of perivitelline space were measured for size (*n* = 20) under digital microscope (AD-7013MZT, Dino-Lite, Anmo, Taiwan) and used in this study as experimental samples.

### Electron microscopy

For transmission electron microscope (TEM) observation, first fertilized egg envelopes were pierced a hole with injection needle and fixed in 2.5% glutaraldehyde in 0.1 M phosphate buffer (pH 7.4) for 12 h at 4 °C. After prefixation, the specimens were washed twice in the same buffer solution for 20 min. and then postfixed in 1% osmium tetroxide solution in 0.1 M phosphate buffer solution (pH 7.4) for 2 h at room temperature. Specimens were dehydrated in ethanol, cleared in propylene oxide, and embedded in an Epon mixture. Ultrathin sections of embedded fertilized egg envelope were taken with an ultramicrotome (Ultracut E, Reichert-Jung, Austria) at a thickness of about 60 nm. The ultrathin sections were mounted onto copper grids, double stained with uranyl acetate followed by lead citrate, and observed with a transmission electron microscope (JEM-1400, JEOL, Japan).

For scanning electron microscope observation, prefixation, postfixation and dehydration were conducted by following the same procedure as that for TEM. The samples were replaced with tert-butyl alcohol and freeze dried (ES-2030, Hitachi, Japan). The samples were coated with Pt by ion sputter (E-1045, Hitachi, Japan). Subsequently, the fertilized eggs were observed under the table top scanning electron microscope (TM-1000, Hitachi, Japan).

## Results and discussion

### Morphology of fertilized eggs

The fertilized eggs formed a mass on the spawning place. The formation of egg mass seems to be a survival strategy because it will prevent being eaten from predator and helpful to move eggs all at once.

**Fig. 1:**
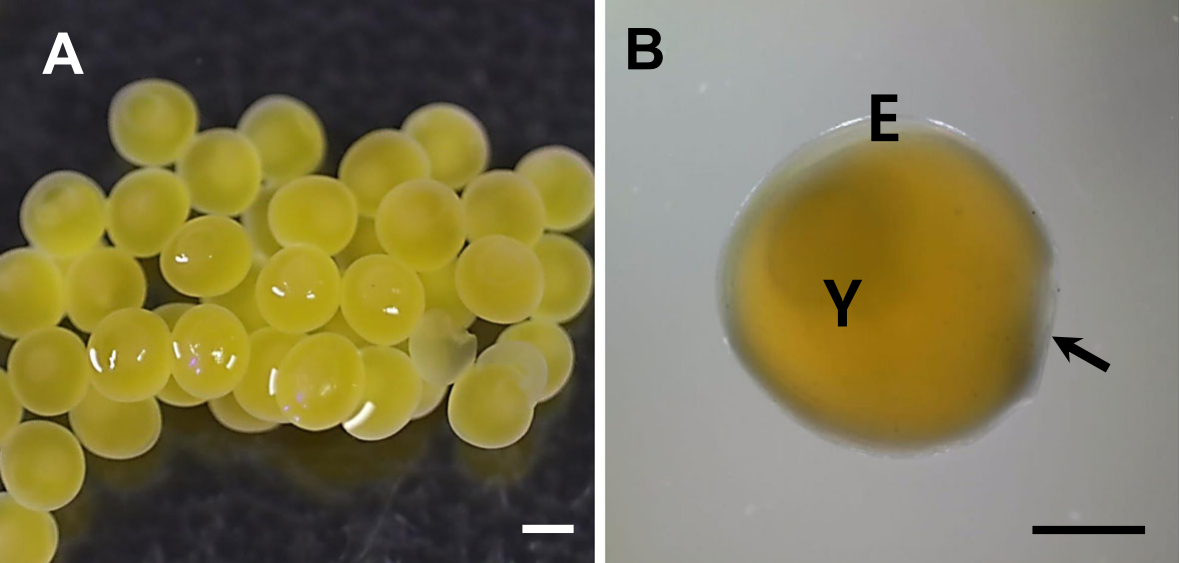
The lumped fertilized eggs (**a**) and a magnified fertilized egg (**b**) of Jumbie teta, *Ancistrus cirrhosus*. E; egg envelope, Y; yolk, an arrow; once adhesive side to form a mass of fertilized eggs (Scale bar = A; 2 mm, B; 1 mm)

And the adhesiveness of fertilized eggs was very strong not only between fertilized egg and spawning place but also between fertilized eggs during spawning (Fig. 1a). But, the adhesive property of fertilized eggs was disappeared after spawning although the adhesive property was maintained in some parts that fertilized egg contact with other egg or spawning place. The fertilized egg of *Ancistrus cirrhosus* was yellowish, spherical, non-transparent, demersal, and has a narrow perivitelline space. There were no adhesive structures (Fig. 1b). The size of fertilized egg was 3.15 ± 0.09 mm (*n* = 20).

In general, morphology of fertilized eggs in fishes is showed family or genus specificities although the size of fertilized eggs and adhesive property are different. The fertilized eggs of fishes belong to Characidae, Belontiidae and Cyprinidae are spherical shape (Kim et al. 1996, 1999, 2005; Joo and Kim 2013). But that of Cichlidae is an oval shape (Deung et al. 1997; Kim et al. 2009). The fertilized eggs of all species belong to Belontiidae have a large oil droplet in center of egg (Kim et al. 1999). The fertilized eggs of all species belong to Belontiidae and Cichlidae are adhesive type, but some fishes belong to Characidae and Cyprinidae are non-adhesive type. Also, fertilized eggs of fishes belong to genus *Nothobranchiu* and genus *Corydoras* have same morphology in same genus (Kwon et al. 2017; Choi et al. 2019). Although tomato clown anemonefish (Pomacentridae) and dark sleeper (Eleotrididae) is different family, the fertilized egg is same morphology, long elliptical shape with a bundle of adhesive filaments (Kim et al. 1998, 2002).

The perivitelline space of fertilized egg of *Ancistrus cirrhosis* was very small enough to stick to egg envelope. Also, the perivitelline space in fishes belong to Cichlidae and Belontiidae is known to small such as *Ancistrus cirrhosis* (Deung et al. 1997; Kim et al. 1999, 2009). The size of perivitelline space is supposed to related with spawning behavior. The fishes belong to Cichlidae and Loricariidae have habit that laying eggs on a spawning ground. And fertilized eggs of Belontiidae have floating property. In fertilized eggs of these species with this spawning behavior or floating property, a large perivitelline space do not need for protection from the external physical impacts. Most of egg scatter such as species belong to Cyprinidae and Characidaeare have a large perivitelline space in fertilized eggs.

### Structure of micropyle

**Fig. 2:**
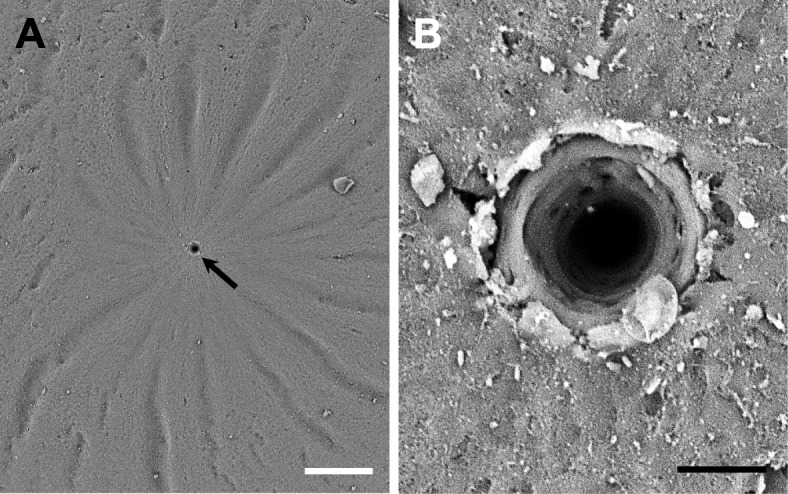
Scanning electron micrographs of a micropyle on the area of the animal pole from fertilized egg of *A. cirrhosus*. The micropyle (arrow) was surrounded by 15–19 furrow lines of egg envelope in a spoke-like pattern (**a**). When magnified by scanning electron microscope, the micropyle was funnel shape (**b**) (Scale bar = A; 100 μm, B; 10 μm)

In most teleost, sperm has no acrosome. Therefore, the egg need a sperm entry site, micropyle for fertilization. In this study, the micropyle was surrounded by 15–19 furrow lines of egg envelope in a spoke-like pattern (Fig. 2a). When magnified by scanning electron microscope, the micropyle was funnel shape (Fig. 2b) and the outer diameter is about 19.3 ± 0.8 μm (*n* = 10). There are no special structures around the micropyle in most teleost, but some species have special structures around the micropyle. The micropyle of *Zacco platypus*, Cyprinidae is surrounded by five peaks of hill structures (Deung et al. 2000). The micropyle of all species belong to Characidae was surrounded by 15–20 uplifted lines of egg envelope in a spoke like structures. This special structure around micropyle shows family Characidae specificity because of all species belong to Characidae have an identical structure (Kim et al. 1996, 2005; Chang et al. 2019). Although *Ancistrus cirrhosis* and species belong to Characidae have similar morphology in spoke like pattern around micropyle, the lines of egg envelope showed different morphology each other. So, we suggest that the special structure, surrounded by 15–19 furrow lines of egg envelope in a spoke-like pattern around micropyle is showed species specificity. The species belong to Belontiidae have same micropyle with funnel shape (Kim et al. 1999) and a plate coral mouth shape in genus *Nothobranchius* (Kwon et al. 2017), but morphology of micropyle is differ according to the species in Cyprinidae (Kim et al. 1998, 2001; Deung et al. 2000). Also, the micropyle was not found on fertilized egg with a bundle of adhesive filaments (Kim et al. 1998, 2002). Consequently, structure of micropyle seems to be family specificity, genus specificity or species specificity.

### Surfaces of the fertilized egg envelopes

**Fig. 3:**
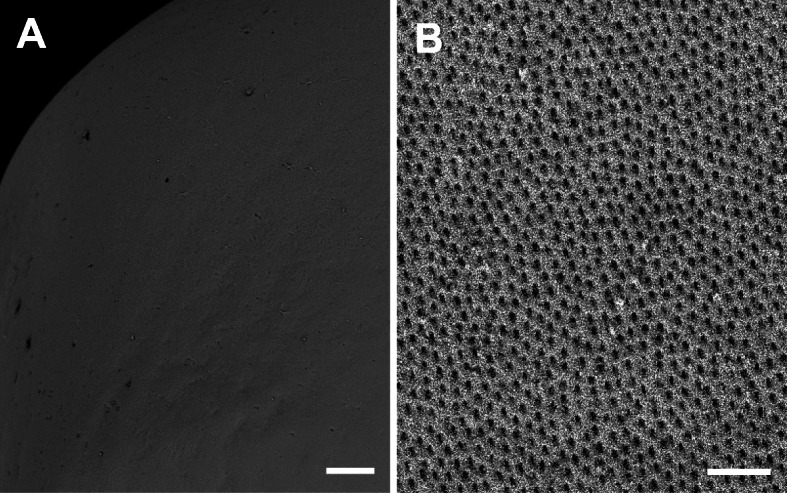
Scanning electron micrographs of outer (**a**) and inner surfaces (**b**) on the fertilized egg envelopes of *A. cirrhosus*. The outer surface is smooth. And the grooves were distributed on the inner surface (Scale bar = A; 100 μm, B; 4 μm)

The outer surface of fertilized egg envelope was smooth (Fig. 3a), but inner surface was rough side with grooves and the grooves were distributed in 3–4 per 4 μm^2^ (Fig. 3b). Smooth outer surface of fertilized egg was found in species belong to other families such as tomato clown anemonefish, Pomacentridae (Kim et al. 1998) and dark sleeper, Eleotrididae (Kim et al. 2002). Also, rough side with grooves in inner surface of fertilized egg envelope is similar with outer surface of fertilized egg envelope of black tetra belong to Characidae (Kim et al. 1996).

The ultrastructure of outer surface of egg envelope is showed genus or family specificities such as *Cichlasoma severum*, *Cichlasoma nigrofasciatum*, *Symphysodon aequifasciatus*, and *Cichlasoma managuensis* belong to Cichlidae (Deung et al. 1997; Kim et al. 2009), *Trichogaster trichopterus*, *Trichogaster leeri* and *Trichogaster trichopterus trichopterus* belong to Belontiidae (Kim et al. 1999), *Nothobranchius foerschi* and *Nothobranchius rachovii* belong to Nothobranchiidae (Kwon et al. 2017), and *Corydoras sterbai* and *Corydoras adolfoi* belong to Callichthyidae (Choi et al. 2019). In Cichlidae, the outer surface was covered with adhesive reticular structures (Deung et al. 1997; Kim et al. 2009), that of Belontiidae have a lot of grooves of envelopes covered by thin adhesive layer (Kim et al. 1999), that of Nothobranchiidae have many adhesive whip-like structures (Kwon et al. 2017), and that of Callichthyidae have adhesive protuberances (Choi et al. 2019).

Most of species belong to Cyprinidae have different structures in outer surface of fertilized egg envelope such as *Tanichthys alborubes* (Kim et al. 1998), *Zacco platypus* (Deung et al. 2000), *Hemibarbus longirostris* (Kim et al. 2001), and *Danio rerio* (Joo and Kim 2013). The outer surface of fertilized egg envelope in *Tanichthys alborubes* have rod-like structures (Kim et al. 1998), that of *Zacco platypus* have Indian club-like structures (Deung et al. 2000), that of *Hemibarbus longirostris* have taste bud-like structures (Kim et al. 2001), and that of *Danio rerio* have knob-like structures (Joo and Kim 2013).

Also, differences of fine structure and the number of process per unit area between *Danio rerio* and *Dnaio rerio* var*. frankei* belong to Cyprinidae could be differentiated by species variation (Joo and Kim 2013). Tomato clown anemonefish belong to Pomacentridae (Kim et al. 1998) and dark sleeper belong to Eleotrididae (Kim et al. 2002) have same smooth outer surface in fertilized egg envelopes. And the adhesive outer surface from *Hemigrammus erythrozonus* are very similar with those of *Gymnocorymbus ternetzi* and *Hyphessobrycon serape,* but have different structure from those of *Hemigrammus ocellifer* and *H. caudovittatus* belong to Characidae (Kim et al. 1996, 2005). These structural characteristics also gives us that outer surface structures can be same even if the species belong to different genus.

### The section of fertilized egg envelope

**Fig. 4:**
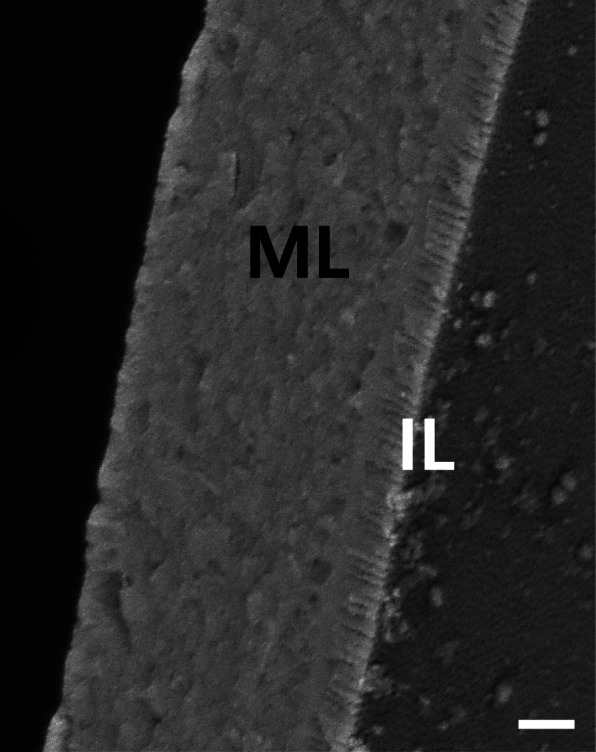
Scanning electron micrograph of the fertilized egg envelopes section from *A. cirrhosus*. The fertilized egg envelope looks like two layers, a middle layer (ML) and an inner layer with microvilli-like pattern (IL) (Scale bar = 5 μm)

**Fig. 5:**
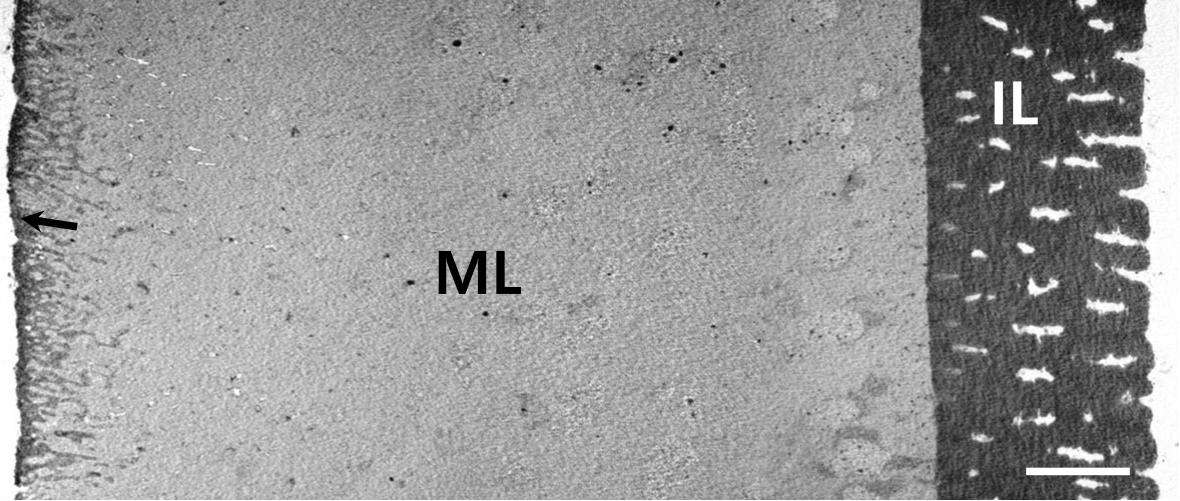
Transmission electron micrograph of section of the fertilized egg envelopes from *A. cirrhosus*. The fertilized egg envelope consisted of three layers, an outer layer with high electron density (arrow), a middle layer with low electron density (ML) and an inner layer with grooves (IL) (Scale bar = 3 μm)

The total thickness of the fertilized egg envelope was about 32.58 ± 0.85 μm (*n* = 20). In scanning electron microscope observation, the fertilized egg envelope looked like 2 layers, homogeneous layer and inner layer similar to microvilli layer in small intestine (Fig. 4). But the fertilized egg envelope consisted of three layers, an outer adhesive electron-dense layer, a middle layer with low electron density, an inner electron-dense layer with grooves under transmission electron microscope (Fig. 5). The unusual structure of inner layer is generally the outer side or a main egg envelope structure in the fertilized egg envelope in teleost. Also, the thickness of fertilized egg envelope was very thick and tough enough to touch the egg with your hands such as contact lens. This is a unique structure of egg envelope in as yet unforeseen structure. So, ultrastructure of egg envelope section seems to be species specificity of *Ancistrus cirrhosis.*

In general, fertilized egg envelope of fish eggs consisted of 2 or 3 layers. The ultrastructure on sections of fertilized egg envelope was same according to the family such as Belontiidae (Kim et al. 1999), Cichlidae (Deung et al. 1997; Kim et al. 2009), Callichthyidae (Choi et al. 2019), and Nothobranchiidae (Kwon et al. 2015, 2017). The fertilized egg envelopes of *Trichogaster trichopterus*, *T. leeri*, and *T. trichopterus trichoperus* belong to Belontiidae consisted of 2 layers, very thin outer adhesive layer and inner layer with grooves (Kim et al. 1999). In the case of Cichlidae, those of *Cichlasoma severum*, *C. nigrofasciatum*, *Symphysodon aequifasciatus*, and *C. managuensis* consisted of 2 layers, an outer layer with adhesive reticular structures and an inner lamellar layer (Deung et al. 1997; Kim et al. 2009). In *Nothobranchius foerschi* and *N. rachovii* belong to Nothobranchiidae, the fertilized egg envelope consisted of 2 layers, electron dense outer layer and an inner lamellar layer (Kwon et al. 2017), and those of *Corydoras sterbai* and *Corydoras adolfoi* belong to Callichthyidae consisted of 2 layers, an outer electron dense layer with protuberances structure and an inner lamellar layer (Choi et al. 2019).

In other species belong to Characidae, the fertilized egg envelopes of *Hemigrammus ocellifer*, *H. caudovittatus*, and *Hyphessobrycon serpae* consisted of 3 layers, and those of *Gymnocorymbus ternetzi* and *H. erythrozonus* consisted of 2 layers. But, the number of the inner layer of egg envelope is different according to the species. Those of *Hemigrammus ocellifer* and *H. erythrozonus* were 3, that of *Gymnocorymbus ternetzi* was 4, that of *H. caudovittatus* was 5, and inner layer of *Hyphessobrycon serpae* consisted of 5–6 layers (Kim et al. 1996, 2005; Chang et al. 2019).

As mentioned above, the number of layers on fertilized egg envelope or section structure are showed species specificity, genus specificity or family specificity. Collectively, these morphological characteristics and adhesive property of fertilized egg, micropyle with spoke-like structure, and ultrastructures of outer surface, inner surface and section of fertilized egg envelope are showed species specificity.

## Conclusions

The structures of fertilized eggs and egg envelope have family or species specificity. The fertilized eggs of Jumbie teta (*Ancistrus cirrhosus*) belong to Loricariidae were yellowish, spherical, non-transparent, demersal, adhesive, and a narrow perivitelline space. But, the adhesiveness of fertilized eggs was disappeared after spawning excluding contact parts. The external shapes and adhesive property of fertilized egg and ultrastructures of micropyle surrounded by 15–19 furrow lines of egg envelope in a spoke-like pattern, and section of fertilized egg envelope with 3 layers, an outer adhesive electron-dense layer with smooth surface, a middle layer with low electron density, and an inner electron-dense layer with grooves in counter structure from other most teleost showed species specificity of *Ancistrus cirrhosis*, differentiated a species from another species.

## Data Availability

No applicable.
